# Identification of an *OsPR10a* promoter region responsive to salicylic acid

**DOI:** 10.1007/s00425-007-0687-8

**Published:** 2008-01-12

**Authors:** Seon-Hee Hwang, In Ah Lee, Se Won Yie, Duk-Ju Hwang

**Affiliations:** 1 National Institute of Agricultural Biotechnology, Rural Development Administration, Suwon, 440-707 South Korea; 2 Department of Molecular Bioscience, School of Biosciences and Biotechnology, Kangwon National University, Chuncheon, 200-701 South Korea

**Keywords:** *cis*-Acting element, *OsPR10a* promoter, Salicylic acid, Salicylic acid induction

## Abstract

*Orysa sativa* pathogenesis-related protein 10a (*Os*PR10a) was induced by pathogens, salicylic acid (SA), jasmonic acid (JA), ethephon, abscisic acid (ABA), and NaCl. We tried to analyze the *OsPR10a* promoter to investigate the transcriptional regulation of *OsPR10a* by SA. We demonstrated the inducibility of *OsPR10a* promoter by SA using transgenic *Arabidopsis* carrying *Os*PR10a:GFP as well as by transient expression assays in rice. To further identify the promoter region responsible for its induction by SA, four different deletions of the *OsPR10a* promoter were made, and their activities were measured by transient assays. The construct containing 687-bp *OsPR10a* promoter from its start codon exhibited a six-fold increase of induction compared to the control in response to SA. Mutation in the W-box like element 1 (WLE 1) between 687 and 637-bp from TGACA to TGAAA completely abolished induction of the *OsPR10a* promoter by SA, indicating that the WLE 1 between −687 and −637 of *OsPR10a* promoter is important in SA-mediated *OsPR10a* expression. We show for the first time that the W-box like element plays a role in SA mediated PR gene expression.

## Introduction

Plants have developed defense mechanisms to recognize pathogens and subsequently activate defense-related genes, such as pathogenesis-related proteins (PR proteins). The major families of PR proteins have been grouped into at least 14 different classes, primarily on the basis of their amino acid sequences (Van Loon and Van Strien 1999). Although the biological and/or biochemical functions of many PR proteins remain unclear, PR2 (β-1, 3-glucanase activity) and PR3 (chitinase) proteins have been shown to inhibit fungal growth (Woloshuk et al. 1991; Sela-Buurlarge et al. 1993). These responses are not limited to pathogen attack and can be induced by defense signaling molecules such as SA, JA and ET (Dempsey et al. 1999; Pieterse and van Loon 1999). To study the defense signaling in plants, many groups have isolated promoters of PR proteins in several plant species, such as *Arabidopsis*, tobacco, pepper, and rice (Malnoy et al. 2003; Hong et al. 2005; Li et al. 2005; Liu et al. 2005a; Lee and Hwang 2006).

Expression profiles of PR proteins, such as *Os*PR1, *Os*PR10 and *Os*PR1b were reported. Originally, *OsPR10a* was known to be induced by probenazole and thus, was called a probenazole-inducible gene, PBZ1 (Midoh and Iwata 1996). Later, PBZ1 was renamed as *OsPR10a* because it shares a similar sequence with (has sequence similarity to) PR-10 proteins. The investigators reported that *OsPR10a* is only induced by probenazole but not by ethephon, NAA, SA, NaCl or mannitol in rice leaves. In contrast, Rakwal et al. (2001) reported that *OsPR10a* is induced by JA, SA, and ABA but not by IAA or GA in light. Ryu et al. (2006) found similar results using RT-PCR. Chen et al. (2006) reported that the elicitor derived from *Magnaporthe grisea* induces *OsPR10a*. However, there has been only one study on *OsPR10a* and *OsCHNIII* promoters, even though many reports are available for the expression profile of PR genes in rice (Rakwal et al. 2001; Hashimoto et al. 2004; Chen et al. 2006; Ryu et al. 2006). The authors reported that *OsPR10a* and *OsCHNIII* promoters are induced by an elicitor derived from *Magnaporthe grisea* by *a* transient assay in vitro. However, the *cis* elements were not analyzed.

Most promoters induced by pathogens or SA contain the W-box, GCC box, RAV1 AAT, or ASF1 motif, etc. (Li et al. 2005; Lee and Hwang 2006; Sohn et al. 2006). Their *cis*-elements have been identified by series deletion of the promoter and site directed mutagenesis of its plausible site. Maleck et al. (2000) analyzed the transcriptome of *Arabidopsis* under defense inducing conditions, and they studied induced promoters such as *PR1*. The W-boxes ((T)TGACC/T) are enriched in the *PR1* regulon promoter. They also described that the W-box like element ((T)TGACA) is also enriched in *PR-1* regulon promoters even though there is no evidence that WRKYs bind to this motif (Maleck et al. 2000).

Transcription factors that can recognize the cognate *cis* element were identified by methods such as the gel-mobility shift assay, yeast-one hybrid, transient assay in plant. WRKY, ERF, RAV, bZIP, MYB, etc. have been shown to be involved in the defense signaling (Rushton et al. 1996, 2002; Eulgem et al. 1999; Kirsch et al. 2001; Heise et al. 2002). The interaction of a transcription factor to its cognate *cis* element is a key step in the process of defense signaling. Among transcription factors, WRKY proteins are the most extensively studied in defense signaling (Eulgem et al. 1999; Robatzek and Somssich 2001; Shimono et al. 2007). Asai et al. (2002) reported that *At*WRKY22 and *At*WRKY29 regulate FLS2-mediated defense signaling. The complex of TGA factor and NPR1 binds to the LS7 in the PR-1 promoter of *Arabidopsis* (Johnson et al. 2003). Furthermore, the TGA/NPR1 complex is as well conserved in rice as in *Arabidopsis* (Fitzgerald et al. 2005). Recently, there are three reports that *Os*WRKY45, *Os*WRKY71 and *Os*WRKY03 regulate the defense signaling in rice, respectively (Liu et al. 2005b, 2006; Shimono et al. 2007), implicating that WRKYs also binds to the W boxes in rice as it does in *Arabidopsis*.

In this study, we analyzed the expression profile of *OsPR10a*. We isolated its promoter and analyzed its *cis*-elements. We also identified the WLE1 (TGACA) controlling induction of *OsPR10a* promoter by SA. This is the first report that the W-box like element actually plays a role in SA-mediated defense signaling.

## Material and methods

### Plant materials

Rice seedlings (*Oryza sativa* cv. Hwachung; seeds from Dr. Wan-He Ye, NIAST, Suwon, South Korea) were grown in a greenhouse at 28°C for 3 weeks. Three-week-old rice seedlings were washed, incubated in tap water for 2 days, and then treated with SA, JA, ethephon, ABA, or NaCl at 1 mM, 100 μM, 100 μM, 100 μM, and 200 mM, respectively. Rice leaves were harvested at the times indicated in the figures. For bacterial inoculations, a strain of *Xanthomonas oryzae* pv *oryzae* KXO98 (*Xoo*; obtained from Korean Agricultural Culture Collection, KACC, Suwon, South Korea) incompatible to *O. sativa* cv. Hwachung was grown in PSA medium (10 g peptone, 10 g sucrose, 1 g sodium–glutamate, and 15 g agar per L) for 2 days and then resuspended in 1 mM MgCl_2 _to a final OD_600_ of 0.5. *Xoo* was sprayed on 3-week-old rice seedlings. After inoculation, plants were incubated in a humidity chamber for 24 h. Samples were taken at the times indicated in the figures and were immediately frozen in liquid nitrogen and stored at −80°C until further analysis.

### Isolation of *OsPR10a* promoter 

Based on an annotation of the rice genome, a −1000 bp fragment of the *OsPR10a* promoter was obtained by PCR from rice genomic DNA using an *OsPR10a* gene-specific primer sets. These primers were designed from the Genbank sequence AL845342. PCR was performed for 30 cycles under the following condition: 94°C for 30 s, 53°C for 1 min, and 72°C for 1 min, followed by a final extension at 72°C for 7 min. The primer sets are as follows:

5′-AAAAAGCAGGCTTGTTTTGAATGCTGGAATGATAA-3′, and 5′-AGAAAGCTGGGTCACTGAAGATATAATCTA-3′.

The underlined sequences match the attB1 and attB2 sites for the Gateway cloning system (Invitrogen, Carlsbad, CA, USA). A 1000 bp amplified PCR product was cloned into pDONR221 to make an entry clone by BP clonase (Invitrogen); successful insertion was confirmed by sequencing.

### Promoter-LUC constructs

The reporter constructs used in the transient expression assays in this study were prepared according to the following procedure. For a 1.0 kb *OsPR10a* promoter, 1000-bp upstream from the start codon of *OsPR10a* was cloned by BP reaction into pDONR221 to make the −1000 bp *PR10a* promoter entry clone described in the previous section. −1000-*Os*PR10a:LUC was created by LR reaction with the −1000-PR10a entry clone and promoter destination vector (attB1-ccdB-Cm^r^-attB2-LUC, unpublished results; Invitrogen). On the basis of W-boxes involved in the activation of defense genes in plants to construct the deleted *Os*PR10a:LUC construct, we amplified the *OsPR10a* promoter region using these sense primers:

−818: 5′-AAAAAGCAGGCTCGTGACATCAGATTGAGTAT-3′
−687: 5′-AAAAAGCAGGCTCGATAAAGGGTATTTGTTTA-3′
−637: 5′-AAAAAGCAGGCTACCTATCATCTAAAAGCATT-3′.

We also used the antisense primer 5′-AGAAAGCTGGGTCACTGAAGATATAATCTA-3′. PCR was performed for 30 cycles under the following condition: 94°C for 30 s, 53–55°C for 1 min, and 72°C for 1 min, followed by a final extension at 72°C for 7 min.

The sequences underlined match attB1 and attB2 sites in the Gateway cloning system. These −818, −687, and −637 bp amplified PCR products were cloned into pDONR221 to make entry clones by BP clonase and confirmed by sequencing. 818-, 687-, and 637-*Os*PR10a:LUC were created by LR reaction with the 818-, 687-, and 637-*Os*PR10a entry clones and the promoter destination vector (attB1-ccdB-Cm^r^-attB2-LUC, unpublished results).

### Site directed mutagenesis

The mutagenized reporter constructs used in the transient expression assays in this study were prepared according to the manufacturer’s instruction (Stratagene, La Jolla, CA, USA). For the mutagenized 687 bp-*OsPR10a* promoter, −1000-*OsPR10a* entry clone (20 μg) was added to 1 μL of 10× reaction buffer, 1 μL (1 ρmole) phosphorylated specific mutagenic primer sets, 0.5 μL 10 mM dNTPs, 0.6 μL Quick solution; and the volume was adjusted to 10 μL with sterile deionized water. The solution was added to 0.3 μL of *Pfu Turbo* DNA polymerase (2.5 units/μL). The reaction matrix mix was used for subsequent PCR. Specific PCRs were performed for 18 cycles under the following conditions: 95°C for 50 s, 60°C for 50 s, and 68°C for 1 min, followed by a final extension at 68°C for 7 min. After generation of the mutgenic double stranded plasmid containing staggered nicks, the product was treated with *Dpn* I and incubated at 42°C for 60 min. The nicked plasmid incorporating the desired mutations was purified with phenol and chloroform extraction and ligated with T4 DNA Ligase at 16°C for overnight. Five microliters of the mutated plasmid was transformed into *E.coli* (DH5α) cells. After transformation, the plasmid DNA was isolated from the mutagenic transformant and confirmed by sequencing. For PCR of mutant strand synthesis reaction, the following mutagenic primer pairs were used: 5′-TGAAATGTAGTCGTACCTATCA-3′ and 5′-TGCTCTGAGATGGGTCTAAACA-3′.

### Particle bombardment and transient expression assays

Leaf bombardments were performed in a Biolistic PDS-1000/He particle delivery system using 1100-p.s.i. rupture disks (BioRad, Hercules, CA, USA). Plasmid DNAs for particle bombardment were prepared as described by the manufacturer’s instructions. For reporters, −1000-bp *Os*PR10a:LUC and 818-, 687-, 637-*Os*PR10a:LUC and m687-*Os*PR10a:LUC were used; 35S:RLUC was used as an internal control to normalize LUC activities between samples after bombardments. About 2 cm lengths of one-week old rice seedlings grown in the dark were cut and incubated on a plate in 1/2 MS medium overnight (Murashige and Skoog 1962). Tungsten particles coated with 1000-, 818-, 687-, 637-*Os*PR10a:LUC, or m687-*Os*PR10a:LUC, and the internal control were delivered into leaf segments by the particle delivery system (BioRad). Leaf segments were incubated at 28°C for 24 h with buffer (1/2 MS medium) or with 1 mM SA and then harvested. Leaf segments were ground in liquid nitrogen and dissociated in 1× passive buffer. The luciferase activities from protein extracts were measured by a dual luciferase system (Promega, Madison, WI, USA) with a luminometer (Aureon Biosystems, Vienna, Austria).

### RT-PCR analysis

Leaf samples were ground to powder in liquid nitrogen, and total RNA was extracted using the Trizol reagent according to the manufacturer’s instructions (Invitrogen). For reverse transcription, total RNA (1 μg) was added to 1 μL of oligo (dT)_16 _and 1 μL of gene specific primer sets (0.5 ρmole); and the volume was adjusted to 15 μL with sterile deionized water. The solution was incubated at 70°C for 5 min, then immediately transferred to ice before the addition of 35 μL of reverse transcriptase master mix containing 10 μL 5× buffer, 3 μL 0.1 M DTT, 5 μL 10 mM dNTPs, 1 μL (200 units/μL) M-MLV RTase (Promega) and 0.2 μL (40 units/μL) RNasin (Promega). The reaction was incubated at 42°C for 90 min before heat inactivation at 65°C for 10 min. Two microliters of each reverse transcriptase reaction was used for subsequent PCR. Gene specific PCRs were performed for 35 cycles under the following conditions: 94°C for 30 s, 53°C for 1 min, and 72°C for 1 min, followed by a final extension at 72°C for 7 min. Samples were visualized on 1.2% agarose gels. For RT-PCR analysis of *OsPR10a* genes in rice, the following primer pairs were used:

***OsPR10a* (D38170)**
- 5′-GCTACAGGCATCAGTGGTCA-3′ and 5′-GACTCAAACGCCACGAGAAT-3′,

***OsActin* (XM469569)**
- 5′-TCCATCTTGGCATCTCTCAG-3′and 5′-GTACCCGCATCAGGCATCTG-3′.

### Generation of transgenic *Arabidopsis*, induction with SA, and fluorescence microscopy

*Os*PR10a:GFP was constructed by LR reaction with pBGWFS7 (Gateway™; Department of Plant Systems Biology, VIB-Ghent University, Belgium) and the *OsPR10a* promoter entry clone described in the previous section, and then transformed into *Agrobacterium tumefaciens* GV3101 for *Arabidopsis*. *Arabidopsis* (Columbia ecotype) was transformed with *A. tumefaciens* GV3101 carrying *Os*PR10a:GFP::GUS and 35S:GFP(35S:pBGWFS7::GUS) as an internal control. A bacterial suspension of *A. tumefaciens* GV3101 carrying *Os*PR10a:GFP::GUS and 35S:GFP::GUS was sprayed on the unopened flowers of *Arabidopsis*. T_1 _plants were screened by 0.3% Barstar spray (Misung, Daejeon, South Korea). Samples were taken from independent T_1_ plants for RT-PCR analysis. T_2_ plants were also screened by 0.3% Barstar spray. Three individual T_2_ plants in each line were used for induction with 1 mM SA at 28°C for 72 h. After induction, transgenic plants carrying *Os*PR10a:GFP and 35S:GFP were examined by fluorescence microscopy using an Olympus SZX-RFL3 (Olympus Optical Co., LTD, Tokyo, Japan). Excitation and emission filters SZX-FGFP and SZX-FGFPA were used for GFP and GFPA (Ex 460–490/Em510- for GFP and Ex460–490/Em510–550 for GFPA). Images were captured with a JP/FV300 camera (Olympus).

## **Results**

### Expression patterns of *OsPR10a* in response to different stimuli

**Fig. 1:**
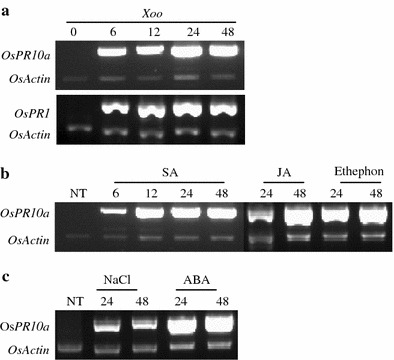
Expression pattern of *OsPR10a* in rice leaves treated with *Xoo* and five compounds. **a** Three-week-old rice seedlings were infected with *Xoo* and were harvested at 0, 6, 12, 24, and 48 h. **b**, **c** Three-week-old rice seedlings were treated with SA, JA, ethephon, ABA, or NaCl and were harvested at 0, 6, 12, 24, and 48 h. Total RNA was isolated from each sample, and RT-PCR was performed using *OsPR10a* specific primer pair. Transcript levels of *OsActin* show that equal amounts of RNA were used in the RT-PCR samples

Several research groups have reported some discrepancy for the expression patterns of *OsPR10a* to some stimuli. Here we looked at the expression of *OsPR10a* by RT-PCR, which is a more sensitive method than reported previously. First, we tested whether *OsPR10a* is induced by a pathogen, as reported previously (Midoh and Iwata 1996; Ryu et al. 2006; Fig. 1a). Induction of *OsPR10a* started at 6 h and reached a maximum at 48 h after *Xoo* infection, as reported previously (Ryu et al. 2006). We also determined whether *OsPR10a* was induced by biotic elicitors such as SA, JA, and ethephon. For the fist time, we show that *OsPR10a* is induced by ethephon (Fig. 1b). Witzh regard to abiotic stress treatments, *OsPR10a* was induced by NaCl and ABA (Fig. 1c). Taken together, we conclude that *OsPR10a* is induced by the pathogen *Xoo*, SA, JA, ethephon, NaCl, and ABA.

### The *OsPR10a* promoter is induced by SA treatment as shown in transient-assay system

**Fig. 2:**
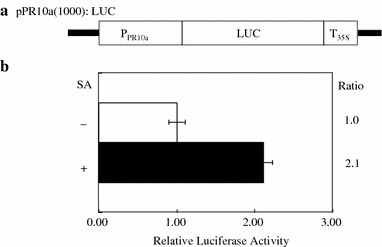
*OsPR10a* promoter activity in response to SA: **a** schematic representation of the *OsPR10a* promoter in the reporter construct. **b** A transient assay showing the *OsPR10a* promoter in response to SA. *Os*PR10a:LUC was bombarded into rice leaves, which were then incubated in MS medium or MS medium containing 1 mM SA at 28°C for 24 h. Protein extracts were made by dissociation in passive lysis buffer as described in “Materials and methods”. Relative luciferase activity is the ratio of the value obtained with the SA-treated *Os*PR10a:LUC divided by the value obtained with the buffer-treated *Os*PR10a:LUC. *Bars* indicate the standard error of three replicates

We analyzed the expression of the *OsPR10a* gene to pathogens and various phytohormones. We focused on the SA-mediated response of the *OsPR10a* gene. To investigate how the *OsPR10a* gene was transcriptionally regulated by SA, we isolated the *OsPR10a* promoter in a 1.0 kb genomic DNA fragment upstream from the start codon of the *OsPR10a* gene by PCR. To analyze whether the 1.0 kb *OsPR10a* promoter was activated by SA as expected by its expression pattern, we carried out a transient assay using particle bombardment. The 1.0 kb fragment of *OsPR10a* promoter was used to make a reporter construct (*Os*PR10a:LUC). Its schematic diagram is shown in Fig. 2a. *Os*PR10a:LUC was introduced into rice leaves by particle bombardment; leaf segments were then treated with either buffer or SA. Protein extracts were prepared from samples after 24 h post-treatment, and their relative luciferase activities were measured. *OsPR10a* promoter activities were expressed as relative luciferase activities. Figure 2b shows a representative graph out of more than three independent experiments. The absolute values from each experiment were different, but the relative ratios from each sample were similar. Luciferase activity in the SA-treated sample was about two-fold higher than in non-treated (control) samples (Fig. 2b). This result indicates that the *OsPR10a* promoter is activated by SA, based on its expression profile.

### The *OsPR10a* promoter is induced by SA treatment in stably transformed *Arabidopsis *

**Fig. 3:**
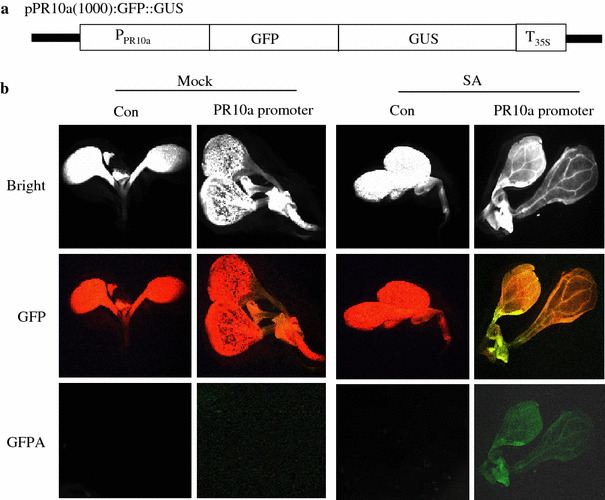
Fluorescence images of *Arabidopsis* transgenic plants carrying *Os*PR10a:GFP: **a** schematic diagram of *Os*PR10a:GFP::GUS fusion construct. **b** Induction of *OsPR10a* promoter by SA. *Os*PR10a:GFP::GUS was introduced into *Arabidopsis* by *Agrobacterium*-mediated transformation. Transgenic *Arabidopsis* seedlings carrying the *Os*PR10a:GFP::GUS was examined using fluorescence microscopy after SA treatment at 72 h. Non-transgenic *Arabidopsis* seedling was used as a control (*left panel* at mock and SA treatments). Shown are the *bright-field* images (*upper panel* Bright), the green fluorescent images using GFP filter (*middle panel* GFP) and the GFPA filter (*bottom panel* GFPA). Images are representatives from two independent experiments. The experiments were repeated at least twice

We further investigated whether SA, as seen in the transient assay, activates the *OsPR10a* promoter using a transgenic approach. A 1.0 kb fragment of the *OsPR10a* promoter was cloned into a promoter-less GFP::GUS expression vector to make a *Os*PR10a:GFP::GUS construct, and was then introduced into *Arabidopsis* by *Agrobacterium*-mediated transformation (Fig. 3a). Transgenic *Arabidopsis* plants (T1) were screened by spraying with 0.3% Barstar and then with self-crossing. Induction of the *OsPR10a* promoter by SA was analyzed by GFP fluorescence in T2 transgenic *Arabidopsis* seedlings treated with either SA or buffer (Fig. 3b). In these GFP filter images (>510 nm), transgenic *Arabidopsis* carrying *Os*PR10a:GFP::GUS exhibited an orange fluorescence in the SA-treated sample because the green fluorescence from GFP was mixed with the red fluorescence from plants themselves (Fig. 3b, middle panel). The green fluorescence from GFP is shown more clearly using a GFPA filter (510–550 nm; bottom panel of Fig. 3b). As shown in Fig. 3b, the *OsPR10a* promoter was clearly activated by SA in transgenic *Arabidopsis*.

### Analysis of *cis*-elements of *OsPR10a* promoter

**Fig. 4:**
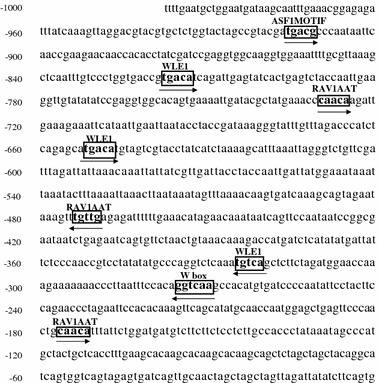
Putative *cis*-acting elements in 1.0 kb *OsPR10a* promoter. The putative *cis*-elements are indicated in boxes and its name is given above each element. *Arrows* indicate the direction of the *cis*-element. *W-box* WRKY transcription factor binding site; *RAV1AAT* RAV transcription factor binding site; *ASF1 motif* bZIP factor binding site; *WLE1* putative WRKY transcription factor binding site

In order to find a *cis*-acting element of the promoter in response to SA, an analysis was done using the PLACE program (a database for PLAnt Cis-acting Elements located at http://www.dna.affrc.go.jp/cDNA/place) (Fig. 4). Among many putative *cis* elements, we only indicated *cis*-elements in boxes known to be related to defense inducers, such as the pathogens, SA, JA, and ethephon, of the *OsPR10a* gene shown in Fig. 1 (Shinshi et al. 1995; Eulgem et al. 1999; Kagaya et al. 1999). The *OsPR10a* promoter analyzed by the PLACE program contains four W-boxes, whose detail sequences are different; there are one canonical W-box ((T)TGACC/T) and three W-box like elements (WLE 1) containing TGAC core (TGACA). There would be more W-box like elements in defense gene regulon promoters. Therefore, we decided to name TGACA as the W-box like element 1 (WLE1). In addition, there are three RAV1AAT elements, and one ASF1 motif element (Fig. 4). The W-box, RAV1AAT, and ASF1 motif are known to be *cis*-elements of the WRKY, RAV1, and bZIP proteins, respectively (Abe et al. 1997; Chen and Chen 2002; Yamamoto et al. 2004). The WRKY, RAV1, or bZIP proteins might be involved in the response of the *OsPR10a* promoter to SA. In addition to them, there are many *cis*-elements involved in ABA responsiveness, even though they are not indicated in Fig. 4. These elements might be involved in the induction of *OsPR10a* by ABA as shown in Fig. 1c. Interestingly, there is no *cis*-element, such as the JA responsive element (JERE) (AGACCGCC) or the ethylene response element (ERE) (AGCCGCC), which is the binding site for ethylene response element binding proteins (EREBP), despite the fact that *OsPR10a* was induced by JA and ethephon.

### Deletion analysis of the *OsPR10a* promoter to identify the regions responsible for the induction by SA

**Fig. 5:**
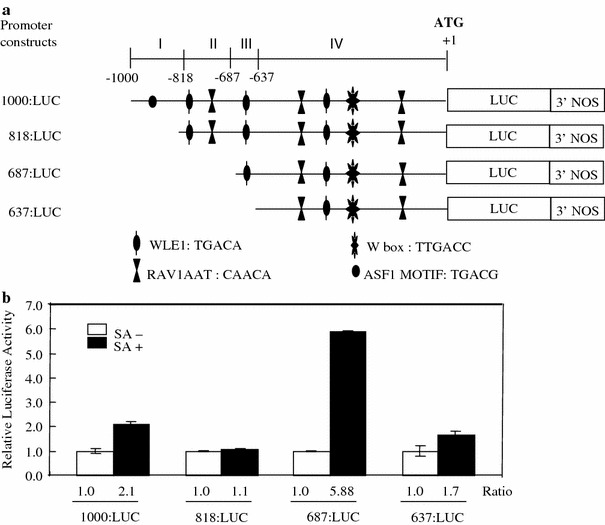
Deletion analysis of *OsPR10a* promoter: **a** schematic diagrams of serial deletion constructs of *OsPR10a* promoter. The numbers to the left of each construct indicate the distance from the start codon ATG. The predicted *cis-*elements ( W-box, RAV1AAT, and ASF1motif) are indicated by their respective abbreviations. The start codon, ATG, is written in *bold*. **b** Luciferase activity in deletion constructs of the *OsPR10a* promoter. Each deletion construct *Os*PR10a:LUC was bombarded into rice leaves, which were incubated in MS liquid medium or MS medium containing 1 mM SA at 28°C for 24 h. Protein extracts were made by dissociation in passive lysis buffer as described in “Materials and methods”. *Bars* indicate the standard error of three replicates. The values are the ratio of the value obtained from each deletion constructs of *OsPR10a* promoter treated with SA or buffer divided by the value obtained from 1.0 kb *OsPR10a* promoter construct treated with buffer

To identify the region of the *OsPR10a* promoter involved in the response to SA, we made serial deletions of the *OsPR10a* promoter by PCR (Fig. 5a). Deletions, beginning with the locations −818, −687, and −637, were fused to the LUC coding sequences and 3′ nopaline synthase gene terminator (Fig. 5a). These four constructs were tested for SA inducibility of the *OsPR10a* promoter by introducing them into rice leaves using particle bombardment and then treating them with either buffer or SA for 24 h. Protein extracts were made from the bombarded leaves and their luciferase activities were measured (Fig. 5b). In the case of the 1.0 kb *Os*PR10a:LUC construct, luciferase activity was increased up to two fold over the control with SA treatment but not in the 818:LUC construct, indicating that there is a weak positive *cis*-element in region I between −1000 and −818 bp of *OsPR10a* promoter (Fig. 5a). One ASF1 motif was found in region I. The exact positive element in this region has not yet been identified. Luciferase activity in the 687:LUC construct was increased up to sixfold with SA treatment, indicating that there is a negative element in region II between −818 and −687 bp of the *OsPR10a* promoter. There is only one WLE1 with the TGAC core (TGACA) and one RAV1AAT element in region II that is known to be bound by transcription factors associated with the defense signaling (Fig. 4). Besides this, there are many putative *cis*-elements in region II (data not shown). Therefore, the exact negative element has not yet been determined. In the 637:LUC construct, there was only about a two-fold increase in luciferase activity with SA treatment, indicating that there is at least one positive element between −687 and −637 bp (region III) and another one between −637 and 1 bp (region IV) of the *OsPR10a* promoter. There is only one WLE1 containing the TGAC core (TGACA) in region III, suggesting that this element may play an important role in the strong inducibility of the 687:LUC construct by SA (Table 1). The W-box, RAV1AAT, and WLE1 are found in region IV of the *OsPR10a* promoter, and at least one of them can act as a weak positive element.

**Table 1:** The list of putative *cis*-acting elements of the *OsPR10* *a* promoter in region III

Region^a^	Position^b^	*cis*-Elements (#)/putative factor^ c^
III	−687 to −637	GT1CONSENSUS(1)/GT-1, WLE1 (1)/WRKY

^a^ *OsPR10a* promoter was divided into four regions depending on the presence of W-box. The region III between −687 and −637 bp of *OsPR10a* promoter

^b^indicates the distance of upstream from the start codon of *OsPR10a*

^c^Putative *cis*-acting elements in region III of *OsPR10a* promoter were analyzed using PLACE (a database for PLAnt Cis-acting Elements located at the web site (http://www.dna.affrc.go.jp/cDNA/place)

### Mutation of a W-box like element in *OsPR10a* promoter abolished its SA inducibility

**Fig. 6:**
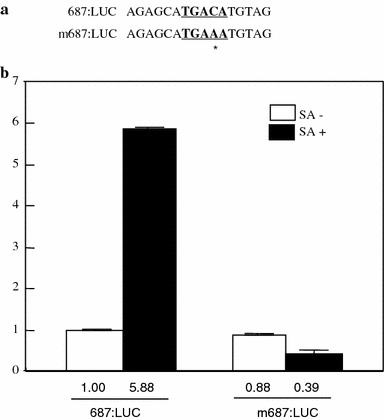
The effect of the mutation in the WLE1 of *OsPR10a* promoter region III. **a** Sequences of the WLE1 (the −659 to −644 bp) in the *OsPR10a* promoter and the mWLE1 with the TGAAA instead of TGACA. The WLE1 sequence is underlined and bolded. The asterisk represents the mutated base in the WLE1. **b** Luciferase activity in 687:LUC and m687:LUC in rice leaves. Bar indicates the standard error of the three replicates

SA inducibility of the 687:LUC construct is the highest among deletion constructs, and only one WLE1 with the TGAC core is present in region III. To further verify this, the WLE1 in region III was mutagenized from TGAC to TGAA (Fig. 6a). Eulgem et al. (1999) reported that the WRKY protein couldn’t bind to a TGAA sequence; therefore, this mutation prevents the association of WRKY to the WLE1 of the *OsPR10a* promoter. Interestingly, SA inducibility of 687 bp-*OsPR10a* promoter was completely abolished in the mutagenized 687 bp-*OsPR10a* promoter, indicating that this WLE1 is involved in the SA inducibility of *OsPR10a*.

## **Discussion**

Plant defense mechanisms to pathogen attack have been extensively studied in *Arabidopsis;* however, it is not well studied in rice. To study the defense mechanisms in rice, we tried to understand the transcriptional regulation of *OsPR10a* because *OsPR10a* has been used as a marker of induction for the defense response in rice (Ryu et al. 2006; Chen et al. 2006). *OsPR10a* was originally cloned by Midoh and Iwata (1996). They reported that *OsPR10a* was induced by *Magnaporthe grisea* and probenazole but not by ethephon, NAA, SA, NaCl, mannitol, and wound. More recently, Rakwal et al. (2001) reported that *OsPR10a* was induced by various phytohormones, such as SA, JA, and ABA, but not by IAA and GA. There were discrepancies between these two reports. In our study, we have shown that *OsPR10a* was induced by *Xanthomonas oryzae* pv. *oryzae*, phytohormones, such as JA, SA, ethephon, ABA and NaCl, but not by IAA and GA. In the case of JA, our result is consistent with the previous report (Rakwal et al. 2001). However, in the case of SA, our result is consistent with the findings by Rakwal et al. (2001), but not with that one by Midoh and Iwata (1996). There might be some differences in the method of SA treatment. We treated rice seedlings with SA by the soil drenching method because our previous result, based on the expression of *OsPR1* gene, indicated that spraying rice leaves with SA does not reliably induce the defense response. In the case of ethephon, our data are also not consistent with the results from Midoh and Iwata (1996). Their data on the expression of *OsPR10a* were generated by Northern blots, whereas our results were generated by a more sensitive method, RT-PCR. Our result is the first report on the response of *OsPR10a* to ethephon. Our data suggest that *OsPR10a* is induced by three different defense signaling transducers (SA, JA, and ethephon). For abiotic stress treatments, *OsPR10a* was induced by NaCl and ABA. In the case of NaCl, our result also differs from the data shown by Midoh and Iwata (1996). We think that there is a sensitivity difference due to the detection methods of *OsPR10a* mRNA between RT-PCR and Northern hybridization as in the case of ethephon. In the case of ABA, our result is consistent with a report by Rakwal et al. (2001). Taken together, we conclude that *OsPR10a* is induced by the pathogens, SA, JA, ethephon, NaCl, and ABA.

In this study, we focused on SA mediated induction of *OsPR10a* because SA mediated defense signaling is the most well studied in *Arabidopsis*. The *OsPR10a* promoter was isolated to study the transcriptional regulation of *OsPR10a* gene. Gene activity was induced by SA in a transient assay system as expected by its expression profile. Chen et al. (2006) reported that it was induced by an elicitor derived from *Magnaporthe grisea* as shown in a transient assay system. SA might be involved in elicitor-mediated defense signaling, yet there was no report on the activity of *OsPR10a* promoter in plants. Our data now have shown that the *OsPR10a* promoter was also activated by SA in stably transformed plants, as we have seen in a transient-assay system.

The *cis*-acting elements of the *OsPR10a* promoter were analyzed to find the elements responsible for its induction by SA. It resulted in many putative *cis*-acting elements. The transcription factors which play an important role in defense signaling are WRKY, ERF, bZIP, MYB, RAV1, etc. (Ruston and Somssich 1998; Singh et al. 2002; Sohn et al. 2006). Therefore, we searched binding sites in the *OsPR10a* promoter for WRKY, ERF, bZIP, MYB, and RAV1. We found W-box, RAV1AAT element, and ASF1 motif element. The W-box, RAV1AAT, and ASF1 motif are known to be the binding sites of the WRKY, RAV1, and bZIP proteins, respectively (Abe et al. 1997; Chen and Chen 2002; Yamamoto et al. 2004). This suggests that the WRKY, RAV1, or bZIP proteins might be responsible for the induction of *OsPR10a* promoter by SA. We also found several ABRE sequences that are known to be responsible for ABA responsiveness of the gene (Shinozaki and Yamaguchi-Shinozaki 1996). This element might be involved in the induction of *OsPR10a* by ABA. There is no *cis*-element, such as JERE or ERE even-though *OsPR10a* was induced by JA and ethephon. Induction of *OsPR10a* by JA and ethephon appears to occur indirectly through some other transcription factors bound to the *OsPR10a* promoter.

Based on *cis*-elements found in *OsPR10a* promoter, three different deletion constructs (818:LUC, 687:LUC, and 637:LUC) were made. Induction of the *OsPR10a* promoter by SA was completely abolished using the 818:LUC construct, indicating that at least one weak positive element exists in region I. In the 687:LUC construct, there was approximately a sixfold increase compared to the 1.0 kb *OsPR10a* promoter construct. This suggests that at least one negative element exists in region II. In the 637:LUC construct, its activity was dramatically reduced compared to the 687:LUC construct, suggesting that there is a positive element in region III. Induction of the promoter by SA was also maintained in the 637:LUC construct, suggesting a positive element is present in region IV. In region III, there were a number of available *cis* elements in the *OsPR10a* promoter (Table 1). However, only one WLE1 with the TGAC core was present in region III. Its nucleotide sequence is different from the canonical W-boxes ((T)TGACC/T) (Maleck et al. 2000). However, they also described that the WLE1 (TGACA) is enriched in *PR-1* regulon promoters. To verify involvement of the WLE1 in response to SA, its sequences were mutagenized (Eulgem et al. 1999). The mutation of the WLE1 from TGAC to TGAA in region III completely abolished the induction of the 687:LUC construct by SA. This suggests that the WLE1 is important in the expression of the *OsPR10a* gene in response to SA. This is the first finding that the WLE1 (TGACA) is important in SA mediated PR gene expression. Interaction of transcription factors and *cis*-acting elements constitute a key step in the defense signaling. The *Os*TGA factor interacts with *Os*NPR1 as reported in *Arabidopsis* (Chern et al. 2001; Yu et al. 2001). These authors suggest that NPR1-mediated defense signaling in *Arabidopsis* is conserved in rice. However, they did not report the identity of the target gene of this complex. Liu et al. (2005b) reported that *Os*WRKY12 induces the expression of *OsNPR1* and *OsPR1b*; however, they did not show evidence that *Os*WRKY12 directly regulates the expression of *OsNPR1* and *OsPR1b* since their experiments utilized transgenic plants over-expressing *OsWRKY12*. Here, we suggest that WRKY may play a major role in SA-mediated *OsPR10a* expression in rice. However, we cannot exclude involvement of other transcription factors in SA-mediated expression of *OsPR10a*. In the near-future, we will carry out electrophoretic mobility assays of the WRKY proteins to the WLE1 described in this study. We will further address what kinds of WRKY proteins regulate the *OsPR10a* promoter and identify the different partners required for SA-mediated *OsPR10a* expression.

## Abbreviations

ABA: Abscisic acid
EREBP: Ethylene responsive element binding protein
ET: Ethylene
GA: Gibberellic acid
IAA: Indole acetic acid
JA: Jasmonic acid
NAA: Alpha-napthalene acetic acid
PR proteins: Pathogenesis-related proteins
SA: Salicylic acid
WLE: W-Box like element 1
*Xoo*: *Xanthomonas oryzae* pv *oryzae*
